# Innovative Strain Sensing for Detection of Exterior Wall Tile Lesion: Smart Skin Sensory System

**DOI:** 10.3390/ma11122432

**Published:** 2018-11-30

**Authors:** Chih-Yuan Chang, San-Shan Hung, Li-Hua Liu, Chien-Pang Lin

**Affiliations:** 1 Department of Civil Engineering, Feng Chia University, Taichung 40724, Taiwan; liuli0063@gmail.com; 2 Department of Automatic Control Engineering, Feng Chia University, Taichung 40724, Taiwan; sshung@fcu.edu.tw (S.-S.H.); chienpang8379@gmail.com (C.-P.L.)

**Keywords:** Smart skin, tile, smart building, public safety, strain gauge

## Abstract

Tiles are commonly used to cover the exteriors of buildings in Taiwan. However, older buildings in Taiwan encounter the problem of tiles falling off due to natural deterioration, which is unsightly, and more importantly, a threat to public safety. Nevertheless, no current method exists that can effectively detect flaws in building tiles in real time. This study combined the fields of civil engineering and automatic control to reduce risks caused by falling tiles by improving real-time detection of at-risk areas. Micro-resistance was combined with fuzzy theory as the logical foundation for evaluating tile status. String-type strain gauges were adopted as sensors to design a smart skin sensory system that could measure signs of deterioration in tile surface lesions. The design was found to be feasible. In the future, it can be further developed for facile real-time assessment of tile status.

## 1. Introduction

Deterioration of building exteriors is a global concern, given the aging of many buildings—particularly the prevalence of high-rise buildings—that could potentially threaten both property and human lives due to events such as earthquakes and climate change [1]. Studies have been conducted in places with climates similar to Taiwan, such as Singapore and Hong Kong, on the problem of tiles falling off of building exterior walls [2,3]. Recently, incidents of ceramic tiles falling from high-rise buildings in Taiwan have become more common, and many cases of injury resulting from such incidents have been reported. According to 2015 statistics, at least 1000 buildings in Taipei have had an incident where tiles fell off [4]. These incidents are caused by buildings aging and tiles gradually degrading due to natural weathering. The number of households in Taiwan was approximately 8.54 million at the end of June 2017, and the average age of buildings was approximately 28 years. In Taipei, old houses (i.e., aged ≥ 40 years) account for 30% of the total number of houses. In terms of regulations governing exterior wall tiles, the Taipei City Government established the Regulations Governing Exterior Wall Safety Diagnosis and Report Management of Buildings in Taipei City (https://s.yam.com/HRGuB, accessed on 7 October 2018) in 2015 to effectively manage and enhance community well-being. The regulations state that buildings that reached a certain age (e.g., 15–30 years), feature risky exteriors, and those that have three floors and above that age between 10–30 years (where the exterior is made of stone curtain walls) should be inspected every six years; buildings that are over 30 years old should be inspected every three years. Owners of buildings must regularly hire personnel from government-approved agencies to conduct pull-out tests, tap tone tests, hanging load tests, and infrared detection tests on exterior walls to evaluate their condition. In addition, to ensure public safety, owners must apply for safety inspections and declare the results. These regulations indicate that people in Taiwan are beginning to understand the importance of technology for diagnosing the status of exterior wall tiles. Common detection techniques adopted include the visual method, tap tone method, and infrared thermography. One study employed pulsed thermography to examine exterior wall tiles, and ultrasonic retests have confirmed its efficacy in detecting deterioration [5]. The tap tone method has also been found to be a favorable approach for diagnosing exterior wall tiles because it takes larger areas into consideration, with different impactors designed to simultaneously measure three different locations, thereby increasing detection efficiency [6]. The study also focused on sound analysis and explored the clustering and classification ability of principal component analysis (PCA), as applied to the impact-acoustics signature in tile-wall inspection with a view to mitigating the adverse influence of surface non-uniformity [7]. Another study proposed the concept of an intelligent facade that automatically regulates wall temperature according to environmental changes, which can reduce health hazards among older adults and children that result from temperature differences [8]. In addition, one study conducted detection by applying passive and quantitative infrared thermography on the exterior wall of high-rise buildings, and the error values were then corrected using algorithms and experiments [9]. There was another way to survey deteration on concrete by robots, such as the research used with the visual-inertial odometry of the robot, the detected flaws of inspection area on the concrete plates are visualized in 2D/3D [10]. Currently, most diagnosis methods require manual operation by humans with the help of instruments. However, because these methods cannot produce real-time detection results when inspecting large areas, their utility in preventive maintenance is limited.

The author conducted a related study on pathogenesis theories in the field of building pathology, where tile deterioration were divided into four onset stages and whether the onset speed was acute was determined. However, the developed strain measurement technique is high cost and its practical applications and economic benefits can still be improved [11]. In this study, a building pathology perspective that considered economics and unfavorable construction-related issues was adopted, and automatic control technology was employed to determine deterioration and installations found on exterior walls of buildings. String-type strain gauge was used to monitor changes in electrical resistance when the exterior walls of a building were deformed due to deterioration, and this deformity was converted into voltage changes by using a signal processing circuit. Finally, smart analysis was conducted according to micro-resistance principles, and fuzzy theory was employed to determine the areas of the exterior walls that were highly deformed, on the basis of which an innovative judgment model was designed. The model was implemented by first testing the string-type strain gauges’ setup and measurements using an acrylic specimen and by observing the characteristic changes in the amount of compression deformation. The feasibility of practical application to the exterior walls of buildings was then established through an experiment with a reinforced concrete (RC) specimen. Verification was performed to determine whether the theoretical values obtained using the acrylic specimen were similar to actual voltage changes obtained in the RC specimen. After comparing the experimental and theoretical values, the correlation between the voltage and tile displacement values was preliminarily confirmed. Decision logic that accounted for the deterioration degree and location of exterior wall tiles was subsequently determined and integrated with the results to form a smart skin sensory system (S4), which is a prototype for a system to be used on real buildings. In the future, this technology could be used as a reference for building components for smart cities that integrate big data and the Internet of Things. Moreover, it could be used in building management to help the development and industrial application of real-time monitoring and analysis for the exterior walls of buildings.

## 2. Materials and Methods

This study used micro-resistance principles as the basis for strain determination. Next, the fuzzy theory was employed to identify the future lesion locations on the exterior wall tiles of buildings. The study was divided into three experiments, which were the acrylic experiment (Experiment A), RC experiment (Experiment B), and S4 experiment (Experiment C). In the first stage, an acrylic specimen was used to investigate the relationship between compression deformation of string-type strain gauges and the voltage value, with the corresponding value of the acrylic deformation obtained by using the acrylic as the substrate wiring. In the second stage, exterior wall compression was tested with an RC specimen to determine the actual correlation between voltage and changes in different tile layers. In the third stage, a lesion location discrimination test was performed on an area of exterior wall tiles (area = 1 m^2^) mounted on a plywood specimen, where the voltage value and fuzzy theory were used to determine lesion locations.

### 2.1. String-Type Strain Gauge

**Figure 1:**
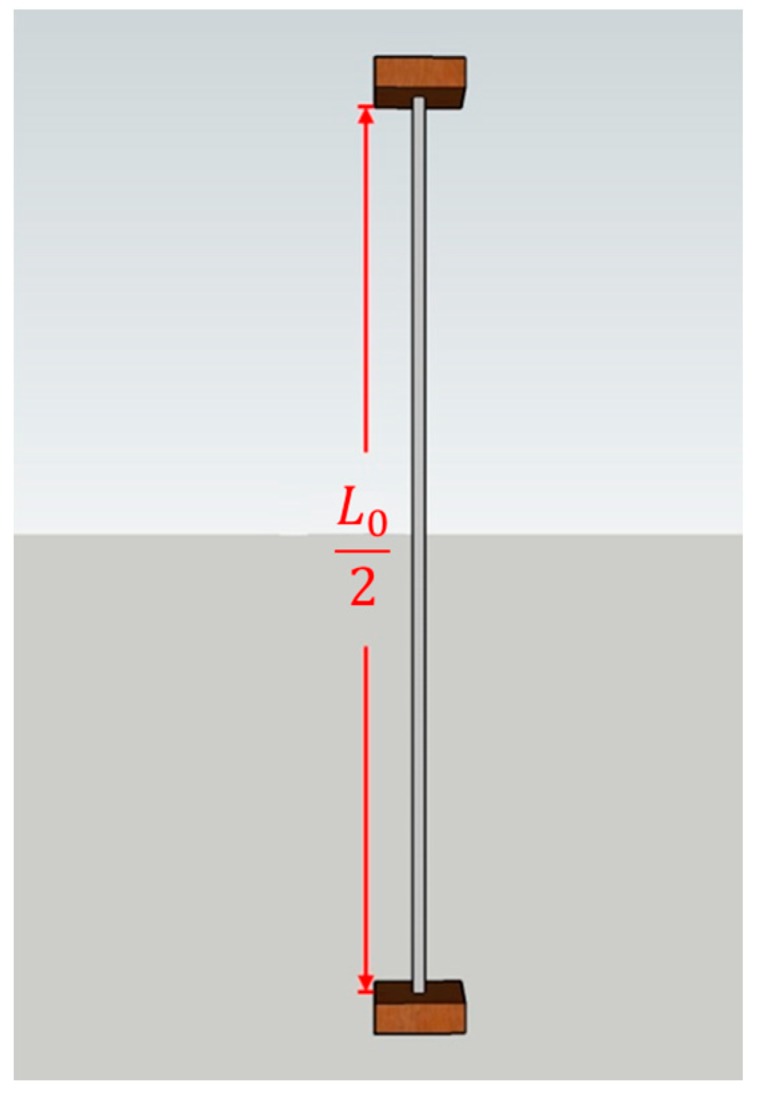
Unstrained wire length.

**Figure 2:**
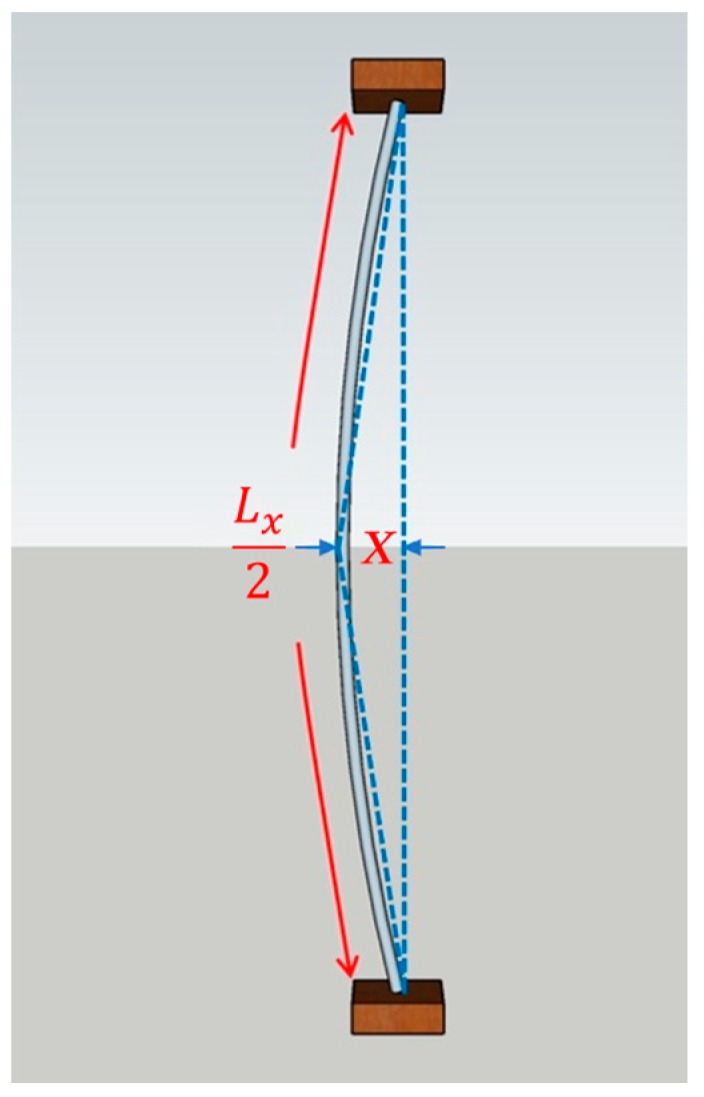
Strained wire length and deformation amount.

**Figure 3:**
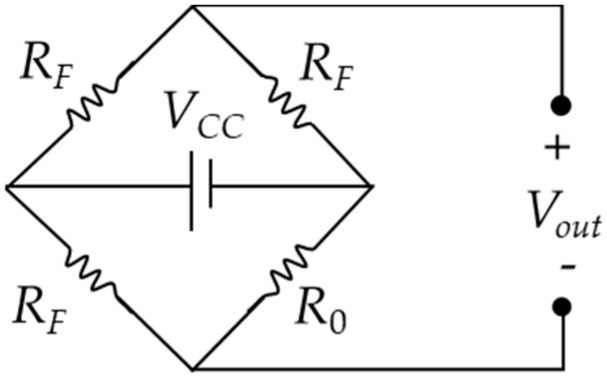
Wheatstone Bridge.

A string-type strain gauge works on the basis of the principles of a resistive strain sensor (or resistive strain gauge). In this study, force was applied to nichrome wires to produce an electrical resistance value, which was then evaluated; resistive strain sensors have great potential for use in human-machine interfaces and so-called electronic skins [12]. A study that employed nichrome wires to measure changes in voltage values generated by the impact of an object was conducted in which zero-voltage correction of the Wheatstone bridge was performed [13]. In the study, nichrome wires were diagonally wired on the surface of test objects. A string-type strain gauge acted as one of the arms of the Wheatstone bridge, wherein the wire length was proportionate to the resistance value. As the nichrome wire was stretched, the corresponding resistance value increased. Equation (1) presents the formula for the resistance value, where *R* is the resistance value, *ρ* is the resistance coefficient, *L* is the wire length, and *A* is the conductor cross-section area. According to Equation (1), as the wire length increases, the resistance value also changes. The setup of the string-type strain gauge is shown in Figure 1, where $L_{0}$ is the original length of the gauge. The folded-back laying method was applied to the wall, and the end point of the wire was set to be on the same side in order to increase the length variation of the string-type strain gauge. Thus, the wire length is $\frac{L_{0}}{2}$, as shown in Figure 2. When lesions on the wall surface produce a deformation of *X* mm, the wire length changes to $\frac{L_{x}}{2}$. The changes to the resistance values applied to the string-type strain gauge before and after compression are governed by Equation (2), where *R*_0_ is the original resistance value of the string-type strain gauge and *R_X_* is the resistance value after compression. Changes in the resistance value caused the Wheatstone bridge to lose balance and produce a change in the output voltage value, *V_out_*, as shown in Equation (3), where *V_CC_* is the power supply voltage and *R_F_* is the fixed resistance of the bridge. The Wheatstone bridge is illustrated in Figure 3. In the present study, because the output voltage of the Wheatstone bridge was extremely small and required processing through an amplifier and a filter, the voltage signal was amplified 2250 times by using a second-order active filter and then 10 times more by using a back-end program. Thus, the voltage signal was amplified 22,500 times (Equation (4)), where *V_O_* is the final output voltage.

(1) $$R≅\rho \frac{L}{A}$$

(2) $$\frac{R_{X}}{R_{0}}=\frac{L_{X}}{L_{0}}=\frac{4\sqrt{\left(\frac{{L_{0}}^{2}}{16}+X^{2}\right)}}{L_{0}}=\sqrt{1+16\frac{X^{2}}{{L_{0}}^{2}}}$$

(3) $$V_{out}=\left(1-\frac{R_{F}}{\sqrt{1+16\frac{X^{2}}{{L_{0}}^{2}}}\times R_{0}+R_{F}}-\frac{R_{0}}{R_{0}+R_{F}}\right)\times V_{CC}$$

(4) $$V_{O}=22500\times \left(1-\frac{R_{F}}{\sqrt{1+16\frac{X^{2}}{{L_{0}}^{2}}}\times R_{0}+R_{F}}-\frac{R_{0}}{R_{0}+R_{F}}\right)\times V_{CC}$$

### 2.2. Experiment Procedure and Apparatus

**Figure 4:**
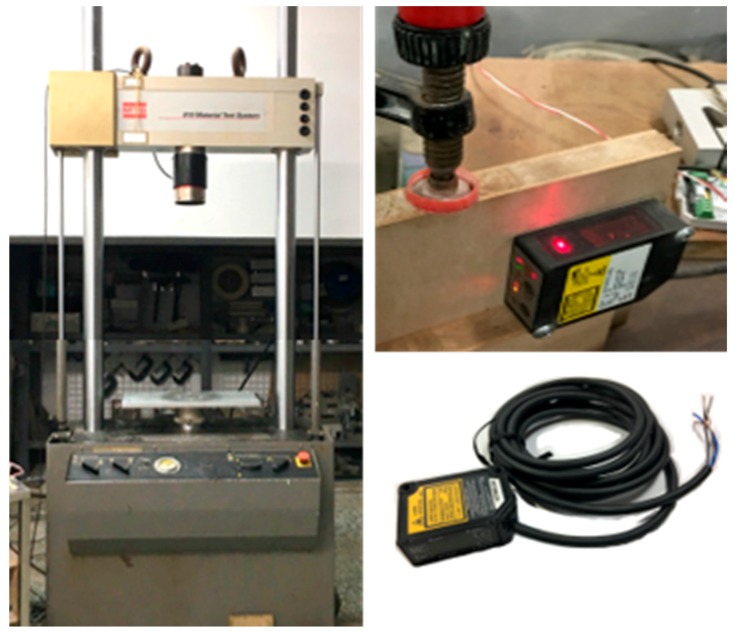
MTS and laser displacement sensor.

The test materials, tile monitoring area, and compressed position are listed in Table 1. In Experiment A, an acrylic specimen was installed using an aluminum frame, and a string-type strain gauge with a tile monitoring area of 30 × 30 cm^2^, which equated to a diagonal length of 42.5 cm (combined length = 42.5 cm × 2 = 85 cm), was installed on top of the acrylic sheet. Next, force was applied to the central point to deform the acrylic sheet. Deformation simulation involved rotating a displacement compression device with one rotation producing a displacement of 1 mm. Each experiment measured the corresponding trend between the voltage value and the deformation amount in 1 mm displacement intervals using multiple regression analysis. Experiment B was an extension of Experiment A using an RC specimen, where string-type strain gauges were arranged in three layers with different lengths during the tile laying process. A 250-kN material test system (MTS) (a dynamic material testing machine, No. 318.25, MTS Systems Corporation, Eden Prairie, MN, USA) was used to compress the center point of the RC specimen to cause displacement (from one to 10 mm in 1 mm intervals). Displacement was measured using a laser displacement sensor (CD22, Optex FA, Ogoto Otsu, Japan) (Figure 4). In Experiment C, a plywood specimen measuring 150 × 150 cm^2^ was installed into an aluminum frame, and tiles were placed on top. The tiles covered an area of 100 × 100 cm^2^, and this area was used to imitate tiles on an exterior wall. Different areas behind the plywood were compressed to cause micro-deformation leading to tile lesion. Micro-deformation was created, as in Experiments A and B, with one rotation producing a displacement of 1 mm, and measurements were again taken from one to 10 mm in 1 mm intervals. Subsequently, S4 was used to detect the deformation position and level of damage caused.

**Figure 5:**
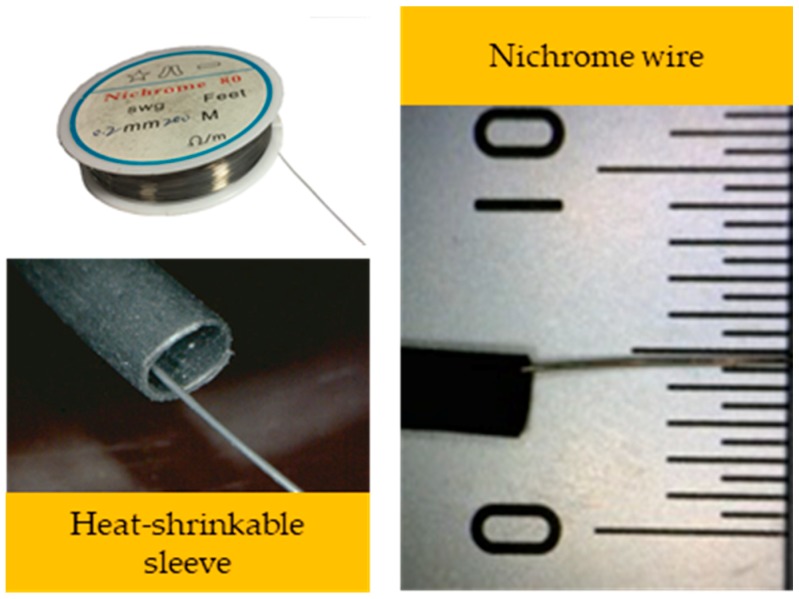
Nichrome wire of the string-type strain gauge.

The string-type strain gauge used in this study employed nichrome wires with a diameter of 0.2 mm that were ductile and displayed a high resistance value of 34.7 Ω/m; this high resistance value made them ideal for signal processing. In Experiment A, a nichrome wire was set on an acrylic sheet. When the acrylic sheet was bent, the string-type strain gauge was compressed, generating a change in the resistance values. In Experiments B and C, heat-shrinkable sleeves were used as outer-layer protection to avoid errors caused by the contamination of the string-type strain gauge (Figure 5). After installing the string-type strain gauges, resistance values were measured using a low-resistance potentiometer. Next, a Wheatstone bridge was used to convert the resistance values into the voltage change amount, and the voltage was inputted into a computer through a data acquisition system (DAQ, USB-6008, National Instruments, Austin, TX, USA) after appropriate amplification and filtering.

### 2.3. Experiments A and B

**Figure 6:**
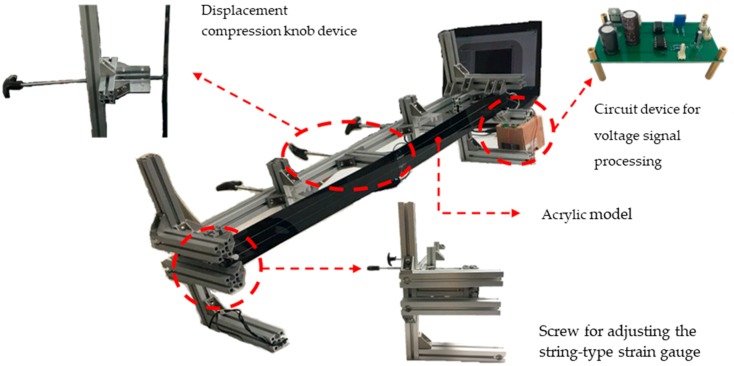
Aluminum frame for the acrylic model experiment.

Experiment A used acrylic as a specimen and had a tile area of 30 × 30 cm^2^ with a string-type strain gauge installed diagonally (combined length = 42.5 cm × 2 = 85 cm). The experiment involved applying a focused force to the center point to cause displacement in order to determine the correlation between deformation of the string-type strain gauge and the voltage value. In this experiment, an aluminum extrusion was employed as the support frame, and the acrylic specimen was clamped at the upper and lower ends. Nichrome wires were placed on top of the acrylic specimen and a securing screw on the frame was used to adjust the tension on the string-type strain gauge. A displacement compression device was installed on the support frame, and the rotating hub of this device created 1 mm of displacement for every rotation, thereby controlling the degree of deformation, as shown in Figure 6. Upon deformation, the string-type strain gauge measured the corresponding voltage value, which was collected by the signal processing circuit.

**Figure 7:**
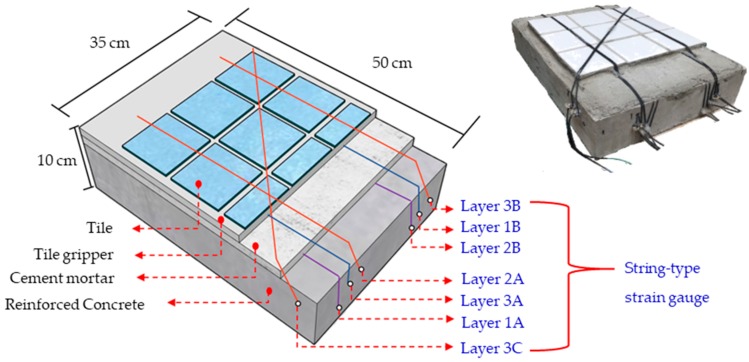
String-type strain gauge layout on the reinforced concrete specimen.

Experiment B used an RC specimen as a mock exterior wall that measured 50 × 35 × 10 cm^3^. A focused force was applied to cause displacement as in Experiment A, an, mmd the tile area was again 30 × 30 cm^2^ with string-type strain gauge installed diagonally (combined length = 42.5 cm × 2 = 85 cm). The string-type strain gauges were installed on the RC, cement mortar, and tile surface layers, divided into Layers 1–3. From Figure 7, the string-type strain gauge laid between the RC and cement mortar layers was designated Layer 1; the string-type strain gauge laid between the cement mortar and tile layers was designated Layer 2; and the string-type strain gauge placed on the surface of the tile layer was designated Layer 3. Layers 1A, 1B, 2A, 2B, 3A, and 3B were arranged in parallel, whereas 3C was arranged diagonally, meaning Layer 3 was arranged both diagonally and in parallel.

### 2.4. Experiment C

**Figure 8:**
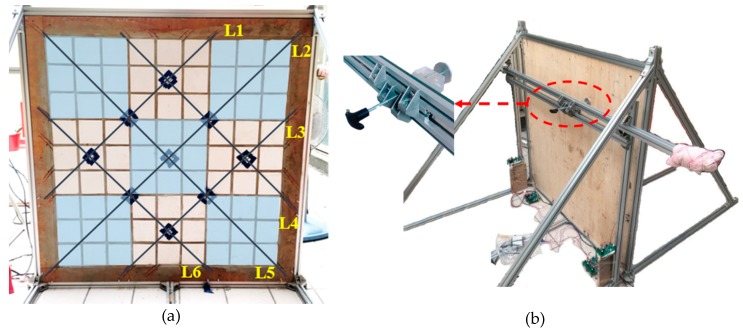
(**a**) Model imitating exterior wall tiles using a plywood specimen and (**b**) a displacement compression device.

Experiment C used a plywood specimen as a model for testing S4 that involved simulating an exterior wall with a tile area of 100 × 100 cm^2^. The tiles were laid on the plywood specimen, which measured 150 × 150 cm^2^, by using a resin tile gripper. Experiment A confirmed the feasibility of applying a string-type strain gauge, and Experiment B demonstrated that fixing string-type strain gauge to the surface of the tile was more economical and convenient for use with buildings. A crisscrossing design was employed to cover the largest area with the minimum amount of string-type strain gauges, with segments folded back and forth by fixing the string-type strain gauges to the same end on the back of the plywood. A total of six strain gauges (L1 to L6) were set on the 100 cm^2^ tile surface, and this surface was divided into nine areas (Areas 1–9), which are visible in Figure 8a. Subsequent system designs were determined according to the nine areas. An aluminum extrusion was used as a support frame for the plywood, and a 5 cm diameter disk was used as a displacement compression device on the rear of the specimen. This device was used to alter the degree of deformation, and the compression position was adjusted by moving an aluminum pole on which the device was fixed. The displacement caused by the device was 1 mm per rotation, as in Experiments A and B (Figure 8b). After deformation of the exterior wall, the voltage values generated by the string-type strain gauges were processed by a signal processing circuit and then converted by the DAQ and input into a computer.

**Figure 9:**
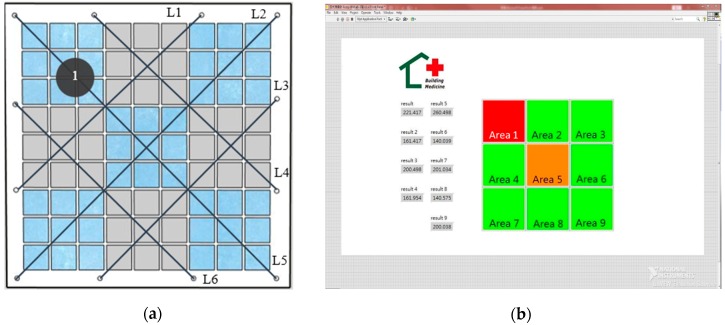
(**a**) Schematic of the compression point 1 and (**b**) area values and hazard levels.

A total of 10 compression points were used. As illustrated in Figure 9a, when the point 1 in a specific area of the exterior wall protruded due to lesion, the string-type strain gauges could measure the changes. S4 was designed on the basis of fuzzy theory using the output voltage values generated by the six string-type strain gauges. Breakdown points and hazard levels were determined by examining the relative magnitude of the voltage values. In Figure 9b, the green, orange, and red areas represent low-risk, moderate-risk, and high-risk areas, respectively. In summary, the S4 system was designed by placing strain on different areas of the plywood and reading the voltage values from the string-type strain gauges. The reference values were determined according to the different effect levels in the different areas of the plywood, from which the breakdown points and hazard levels could be determined.

## 3. Results

### 3.1. Results of Experiment A

Experiment A used an acrylic specimen and comprised 600 measurements—voltage values were measured 10 times for each displacement value from one to 10 mm for one set of measurements; a total of six sets were conducted for a total of 600 measurements. This study set the error value at 0.05 and proposed the following null and alternative hypotheses: The null hypothesis was “compression distance did not have an effect on voltage value,” and the alternative hypothesis was “compression distance had an effect on voltage value.” Subsequently, six sets of numerical analyses were performed using polynomial regression to obtain six regression curves and formulas *y*1 to *y*6.

**Figure 10:**
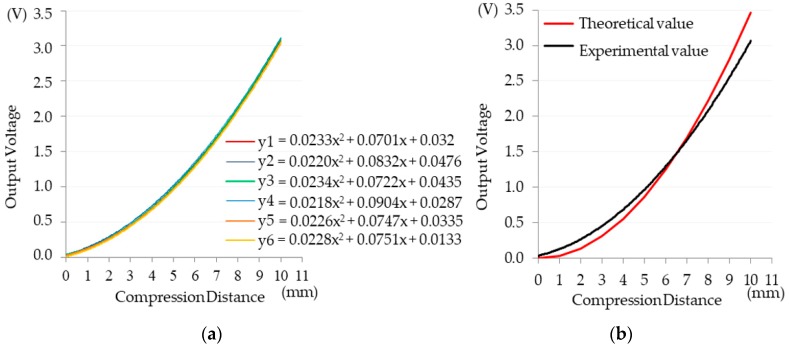
(**a**) Result analysis for Experiment A and (**b**) comparison between theoretical and experimental values in Experiment A.

The slopes of the six trend lines fell within the range of 0.021–0.024, and the trend direction was consistent, as shown in Figure 10a. In the regression analysis, the coefficient of determination (*R*^2^) was used as the index. The *R*^2^ of the six regression lines exceeded 0.9990, indicating that displacement had a great influence on the voltage values during the displacement process. The trend line revealed that deformation of the nichrome wire on the acrylic sheet caused a rise in the corresponding measured voltage. The value for each compression point in Figure 10a is the average of 60 data points, and the curve is the polynomial curve of 10 points regressed from one to 10 mm (*y* = 0.0233*x*^2^ + 0.0701*x* + 0.032, *R*^2^ = 0.9994). According to the results plotted in Figure 10b, the theoretical and experimental values displayed a similar trend, indicating that the experiment had validity, and the use of nichrome wires as string-type strain gauges is feasible and reliable.

These findings indicate that, as the exterior materials of a building deformed, a string-type strain gauge would also deform and produce a change in resistance values. In Experiment A, an acrylic specimen was used instead of an actual wall to measure the relationship between compression of a specimen and output voltage and to confirm the reliability and validity of using a string-type strain gauge. The feasibility of using a string-type strain gauge to measure deformation of an exterior tile wall was confirmed and served as a reference for the subsequent experiments in this study. In the future, the methods of this model may be applied by sensory systems made from other materials to detect deformation on the exterior tiles of buildings.

### 3.2. Results of Experiment B

**Figure 11:**
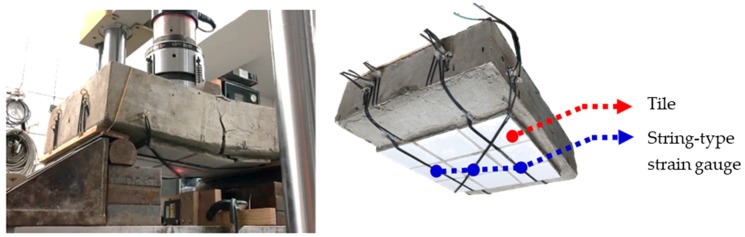
Compression test that used the MTS to apply force on the back of the specimen.

**Figure 12:**
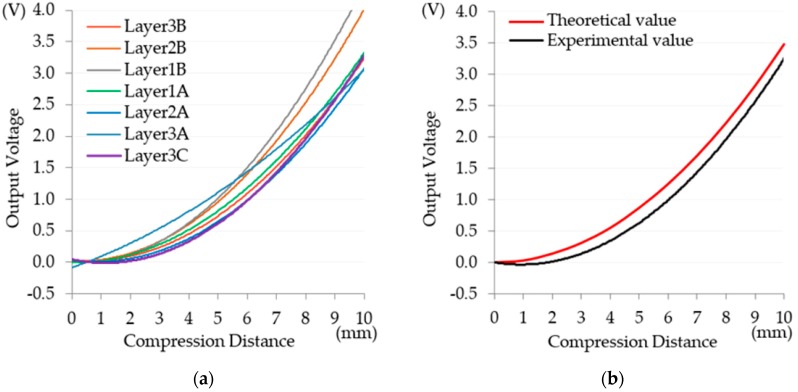
(**a**) Result analysis for Experiment B and (**b**) comparison between theoretical values and experimental values of Layer 3C.

In Experiment B, using an RC specimen, a 250-kN MTS (No. 318.25, MTS Systems Corporation, Eden Prairie, MN, USA) was employed to compress the specimen and generate displacement. The use of an MTS is common in displacement tests for RC [14,15] because deformation is generally a long-term process, and the use of an MTS, which forces protrusion, can shorten the required time. Displacement was measured using a laser displacement sensor to verify the feasibility of using string-type strain gauges on the exterior walls of buildings, and the optimal laying level was identified by comparing the experimental values with the theoretical values. The string-type strain gauges were laid in three layers—the concrete, cement mortar, and tile layers (Layers 1–3, respectively)—with seven gauges across the three layers. As in Experiment A, force was applied to a single point on the back of the specimen using the MTS (Figure 11), at displacement values of one–10 mm, and the voltage values of the seven gauges were measured simultaneously during the compression process, providing seven sets of values for polynomial regression. Each gauge had 11,992 measurements. The seven regression curves are presented in Figure 12a, and reliability was indicated by an *R*^2^ > 0.98. Subsequently, the performance of the string-type strain gauges across the three layers was compared and analyzed. A string-type strain gauge comprises a fine wire, which makes it convenient for laying on the surface of a tile during construction and maintenance. Consequently, the wiring method in Layer 3C was the optimal design, because its polynomial regression equation was *y* = 0.0419*x*^2^ − 0.0965*x* + 0.0444, and its *R*^2^ was 0.9972. Comparisons between the theoretical and experimental values showed that the trend for the compression distance was consistent with the voltage values, with the difference between the two curves being almost equidistant, indicating that the results have a certain degree of validity (Figure 12b).

This experiment imitated the actual conditions of an exterior wall and different layers of string-type strain gauges were installed on RC specimens. MTS was used to compress and record the changes, and the data of the string-type strain gauges of different layers were collected. The analysis results revealed that the string-type strain gauges in each layer were all feasible. However, for convenience during construction and maintenance, the string-type strain gauge on the outermost tile surface was chosen as the basis for Experiment C. Nonetheless, this experiment demonstrated that data values can still be effectively measured in the concrete and cement mortar layers using string-type strain gauges with heat-shrinkable sleeves, and this information can be used by researchers as a reference for future experiments.

### 3.3. Results of Experiment C (S4)

During the experiments in this study, changes in the resistance values of the string-type strain gauges were extremely small and difficult to determine directly. Therefore, a conversion circuit was used to convert the changes in resistance values into voltage signals; the Wheatstone bridge is the most commonly used resistance conversion circuit and one that is the most suitable circuit for measuring small resistance changes [16]. However, commercially available resistors often contain errors. Even if four 1-KΩ resistors with the same specifications are installed on the four arms of a Wheatstone bridge, the output voltage may not be zero volts. In a high-magnification amplifier circuit, such an error may cause the amplifier to export saturation voltage, meaning that an accurate value cannot be measured. Therefore, in this study, error compensation was performed for the Wheatstone bridge. In addition, when electric current flows through a string-type strain gauge, heat energy causes the temperature to rise. This rise in temperature positively affects the resistance values of a string-type strain gauge, which results in an unbalanced Wheatstone bridge. Such a situation can lead to erroneous belief that the gauge is strained. Therefore, temperature compensation was performed; both the aforementioned compensations minimized the chance of misjudgment in this experiment.

**Figure 13:**
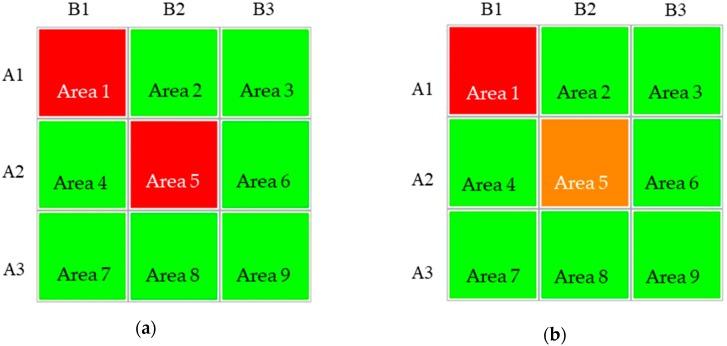
Warning areas judgment logic. (**a**) First Step; (**b**) Second Step.

Fuzzy theory was used as the foundation for the design of S4, wherein the measured voltage value was transmitted to the computer, and the fuzzy theory was applied to determine the location from which deformation or deterioration occurred. One study used LabVIEW for software control of complex devices and DAQ to perform laser imaging [17]. In the present study, LabVIEW (LabVIEW 2015, National Instruments, Austin, TX, USA) was employed as the human-machine interface for S4 that can be used by relevant personnel for monitoring. S4 conducts location judgment and hazard warning by determining the relative magnitude of the voltage value, and colors are displayed on the system to distinguish the relative risk. For example, S4 first uses green to indicate a safe area and red a dangerous area, after which it delineates green as low-risk areas, orange as moderate-risk areas, and red as high-risk areas. The system then investigates the relationship between these locations. The experiment was conducted according to the 10 preset deformation points. As illustrated in Figure 13a, more than one hazard area was identified because the values calculated were relatively high during the first round of judgment by S4. If the system revealed two red hazard areas but only a single compression test was performed in the upper-left corner of Area 1, which may be due to inaccuracy caused by change in the string-type strain gauge or large damage intervals for the numerical values. According to the logic of the system, a second-step judgment is conducted based on the correlation values between the locations to more accurately determine the hazardous areas. The method involves adding up the connected areas (intersected horizontally and vertically) in each area and arranging the rows (A1, A2, and A3) and columns (B1, B2, and B3) into nine squares. Take A1 and B1 as examples; A1 represents the sum of the values of Areas 1, 2, and 3, and B1 represents the sum of the values of Areas 1, 4, and 7. Take Area 1 as another example; Area 1 is the sum of the values of row A1 plus column B1 (Areas 1, 2, and 3 + Areas 1, 4, and 7). Larger values are normally caused by the fuzzy theory or abnormal voltage values. The most hazardous areas are more accurately presented after the first and second steps have been taken (Figure 13b).

**Figure 14:**
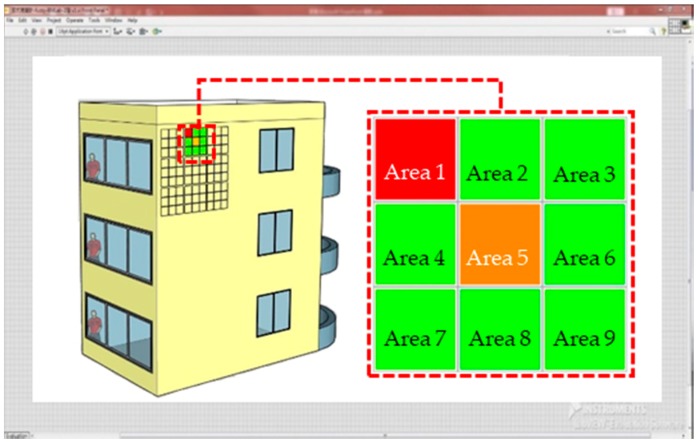
S4 monitoring screen.

In future real-world applications of S4 on the exterior walls of buildings, the system could display hazard risks according to a color gradient on the rear management system through real-time monitoring with ranges for each floor, direction, and building, or predictions for damaged areas. The hazard level on the current system works through color stratification, from low risk (green), to medium risk (orange), through to high risk (red) (Figure 14). In the future, investment in the real-time monitoring feature of S4 could enable building managers to understand the condition of exterior wall tiles on their building, thereby facilitating planning of tile maintenance. If the system exhibits slight color changes during the day before returning to green, it may be caused by thermal expansion of the tiles during the heat of the day. In such a situation, follow-up observations are recommended. The system monitoring uses intermittent polling, which means that the frequency of the measurement can be adjusted. To save power consumption and related costs, selecting only one of the multiple input signals to be transmitted to the output circuit at one time is recommended. Under these conditions, all the string-type strain gauges can be measured, respectively, in the allowable reaction cycle time to reduce the costs of circuit dissipation.

## 4. Discussion

Experiments A and B (using acrylic and RC specimens, respectively) provided experimental results with values that shared same trends as those obtained theoretically, which indicated that string-type strain gauges are feasible for use in monitoring lesions in exterior wall tiles. Nichrome wires are usually used as electric heating elements, and those available on the market are bare wires, which makes them susceptible to wind and temperature changes. Future studies with higher budgets could employ nichrome wires with enamel covers to reduce disturbances and make the wires more durable, thereby improving data stability. Experiment C employed a plywood specimen as a mock exterior wall, with tile displacement controlled from the rear and voltage values examined to determine deformation. However, a few of compression points led to poor results in subsequent experiments, and several limitations were identified for Experiment C. First, the plywood specimen was unable to withstand multiple compressions, in which the various experiments caused it to deform, resulting in the data for subsequent compressions being relatively unstable, as compared with the data for the initial compressions. Second, the experimental budget was limited, and with the cost of each specimen exceeding US $350, for Experiment C, only the feasibility assessments could be made beforehand due to limited budget. In the future, a higher budget, improved experiment site, and longer experiment are required to obtain more complete experimental data in order to determine system stability and conduct long-term monitoring or active-sensing experiments. Nonetheless, this study has provided initial experience and evidence regarding the feasibility of string-type strain gauges for use on exterior tile wall surfaces, which shall diminish errors observed in experiments and actual practice. Finally, four major discussion points were identified throughout this study: Tension value setting, string strain gauge mounting and technology, lesion determination using S4, and future applications.

1. Regarding the tension value setting, experimental errors occurred because the analyzed trend line of the experimental results did not perfectly match each other. After investigation, errors during the experiment were identified. These arose because the nichrome wires were bare wires, which could easily be affected by wind and other disturbances. This led to errors in the resistance values of the string-type strain gauges. Therefore, future research should examine the tension value. In this study, the initial tension value of the gauges was measured before the experiment. However, the value changed from the initial value after conducting several experiments. In the future, the tension values must be remeasured or the string-type strain gauge replaced after compression. The present did not do this due to the difficulty of constructing the specimens and budgetary constraints. In the future, the different tensions of string-type strain gauges must be verified to obtain the optimum value (uniform tension) before using them. Moreover, when tile lesions occur, string-type strain gauges must be replaced, or under non-replaced circumstances, to measure the string-type strain gauges’ tension values.
2. Regarding the technology and installation of strain gauges, a study used ultrasonic technology on P(VDF-TrFE) thin films in order to make the thin films vibrate at high frequencies. Thin film deformation resulted in a change in the resistance values, and the voltage resonance on the thin films was used to estimate piezoelectric coefficients [18]. With regards to the materials used in strain gauges, a study noted that nanocomposites exhibited outstanding temperature compensation capability and sensitivity [19]. Existing piezoelectric sensors have the advantages of being low cost and having high levels of sensitivity and stability, which means that they are suitable for assessing small areas. However, a substantial number of piezoelectric sensors are required to assess a large area. Therefore, the present study employed string-type strain gauges that could detect extremely stable electrical signals. However, string-type strain gauges are easily influenced by outdoor environments. In consideration of this phenomenon and to increase installation convenience, nichrome wires were used in the strain gauges and to serve as temperature compensation in the Wheatstone bridge. To prevent deterioration of the string-type strain gauge mountings, heat-shrinkable sleeves were used as isolators at the intersection of two string-type strain gauges. In the future, enameled wires are recommended to isolate string-type string gauges from external deterioration factors. The deterioration factors of string-type strain gauges are primarily external forces and humidity, with external forces being environmental forces such as wind and rain, rather than damage caused by humans or birds. Humidity contributes to the rusting of string-type strain gauges, which destabilizes their resistance values. If enameled wires are employed in future revisions, humidity can be prevented from entering the string-type strain gauges, which would prolong their lifespans considerably. Additionally, the fixed end of the string-type strain gauges could be fixed on the gap between tiles. This installation would avoid destroying the decorative materials.
3. The lesion determination method in S4 has revealed the possible influences on tile displacement. In addition to physical damage, other factors such as temperature differences between night and day may have an influence. In Experiment A, the voltage value was zero when the compression distance was zero. Therefore, in S4, fuzzy theory was applied to judge the relative position of the string-type strain gauge and verify the calibration accuracy of the warning system. If the warning light in S4 is on and the voltage value returns to zero within a day, the situation is considered normal shrinkage of the tile and its structure; if the voltage value is large and maintains at a high value or continues to increase, it may be a sign of tile lesion, in which case, further checks should be performed to determine whether it is a result of a tile hollowing, protruding, or falling off.
4. The innovative system designed in this study, S4, demonstrated feasibility for use in future applications. However, the experiment in this study only imitated deterioration of an exterior wall, and therefore the findings may differ from those in a real-world situation. In the future, a string-type strain gauge should be installed on a real exterior wall to conduct long-term measurement and see how measurement naturally deteriorates in order to adjust the output value change for benchmarking. Regarding tile replacement and maintenance, the cost of system installation and tile-falling situation were considered in this study, and thus wiring was not installed on every wall tile, but was laid using a crosslinking method. Consequently, S4 provides warnings for tile areas rather than individual tiles, and users of this system are recommended to conduct replacement and maintenance work by tile areas. Regarding application of data and algorithms, a study used a database for equipment training. In that study, new images were merged with the training set to develop a multiagent system for classifying the gender and ages of people in images [20]. In another study, an artificial neural network was utilized to determine energy optimization plans for improving the energy conservation performance of office buildings [21]. We advise that researchers conducting future studies on this topic use database training and improved algorithms to further the precision of their systems. For example, they may use supervised learning with artificial intelligence to continuously adjust the propagated weight in a network, thereby reducing the gap between the output and expected values, improving the precision of the evaluation results, and increasing the benefits of implementing the system.

## 5. Conclusions

Diagnosis of lesions in exterior wall tiles has previously required on-site examination, such as through infrared thermography, the tap tone method, or the use of robots; however, all these methods cannot easily provide real-time warnings. In this study, string-type strain gauges were applied alongside fuzzy theory to create an innovative system, S4, to prevent lesions in building exterior wall tiles and plan maintenance in advance. Taiwan has many buildings that use tiling as a cladding material on exterior walls. In the future, different wire stocks could be considered for use in S4 once the system is more accurate and stable. The types of cladding tiles used on exterior walls are diverse, and subsequent studies could examine different exterior cladding materials to discover the optimal wiring methods and fuzzy theory for each type of material. Researchers can refer to the findings of this study—for example in terms of tension value setting, lesion determination using S4, and experimental limitations—to continue and refine this research direction. Introduction of neural networks for real-time monitoring is recommended for future applications, which could help building managers with more efficient real-time monitoring and help ensure public safety.

## Figures and Tables

**Table 1:** Experimental information.

Experiment	Specimen	Tile Monitoring Area	Compressor Position
A	Acrylic	30 × 30 cm^2^	Center point of the string-type strain gauge
B	Reinforced concrete	30 × 30 cm^2^	Center point of the RC specimen
C	Plywood exterior wall	100 × 100 cm^2^	At 10 irregular points behind the plywood
